# A five-year review of necrotising fasciitis in a tertiary referral unit

**DOI:** 10.1308/003588413X13511609956093

**Published:** 2013-01

**Authors:** RA Swain, JC Hatcher, BS Azadian, N Soni, B De Souza

**Affiliations:** ^1^Imperial College London,UK; ^2^Chelsea and Westminster Hospital NHS Foundation Trust,UK

**Keywords:** Necrotising fasciitis, Abscess, Fasciitis, Sepsis, Infection

## Abstract

**Introduction:**

Necrotising fasciitis is a life-threatening illness that is often difficult to diagnose. Immediate debridement and intravenous antibiotic therapy are required to limit the spread of infection. This five-year audit aimed to review the number and outcomes of all cases of necrotising fasciitis admitted to a tertiary referral unit and to assess the validity of the Laboratory Risk Indicator for Necrotising Fasciitis (LRINEC) scoring system.

**Methods:**

A retrospective analysis of patient notes over the five-year period from October 2006 to October 2011 was undertaken. The LRINEC score was calculated for each patient to evaluate its usefulness.

**Results:**

Overall, 15 patients were diagnosed with necrotising fasciitis. Three patients died. The median age of patients was 51.0 years (range: 34–76 years). There were no obvious predisposing factors in 8 cases but patients had a median of 2.0 co-morbidities. The most common infective agent, present in five patients, was Group A *Streptococcus*. Other monomicrobial agents included Group G *Streptococcus* and *Klebsiella pneumoniae*. Polymicrobial infections were less common than monomicrobial infections and two patients had a polymicrobial infection including methicillin-resistant *Staphylococcus aureus*. Although the LRINEC scoring system identified 12 of the 15 patients as having a high or intermediate likelihood of necrotising fasciitis, 3 were classified as low likelihood.

**Conclusions:**

This limited case series strongly suggests that the LRINEC system is too insensitive for diagnosis.

Necrotising fasciitis, the notorious ‘flesh-eating bug’, was first described formally 60 years ago.^1^ Swift, aggressive management has been shown to produce the best outcomes.^2^ To aid diagnosis, the Laboratory Risk Indicator for Necrotising Fasciitis (LRINEC) scoring system^3^ was devised to stratify the likelihood of infection in patients on presentation (Table 1). A score of 5 or under is low risk (ie there is less than a 50% chance that the infection is necrotising fasciitis). A score of 6–7 is intermediate risk and 8 and above signifies a high risk (more than 75%) that the patient is suffering from necrotising fasciitis.

Few case series from the UK have been reported in recent years so this case series was carried out to ascertain the number, characteristics and outcome of cases from a tertiary referral unit over a five-year period. The validity of the LRINEC scoring system was also evaluated.

## Methods

This was a retrospective review of the medical records of patients who developed necrotising fasciitis during the period between October 2006 and October 2011 at a tertiary referral unit in London. A diagnosis of necrotising fasciitis was made based on clinical findings (such as erythema and pain out of proportion to physical appearance) and confirmed on surgical investigation revealing grey necrotic tissue, lack of dermal bleeding, lack of resistance on blunt dissection and foul ‘dishwater’ pus. The LRINEC scoring system was assessed retrospectively to evaluate if it would have been helpful.

## Results

### Patient demographics

There were 15 patients (10 male, 5 female) with an age range of 34 to 76 years (median: 51.0 years). There were no obvious predisposing factors in eight of the cases. Two patients had suffered minor burns and another may have sustained an insect bite. One patient presented to the accident and emergency (A&E) department three days after an inguinal hernia repair. Two patients developed the infection while in hospital after surgery, for one it was 9 days following an elective panproctocolectomy and for another it was 11 days after ankle fixation. Of the 15 patients, 12 had significant co-morbidities (Table 2), with a median of 2.0 co-morbidities per patient (range: 0–8). These included iron deficiency anaemia, hepatitis B and rheumatoid arthritis. One patient had human immunodeficiency virus. Four of the patients smoked or had recently stopped smoking.

**Table 1:** Laboratory Risk Indicator for Necrotising Fasciitis scoring system^3^

Parameter	Range	Score
C-reactive protein	>150mg/l	4
White cell count	<15/mm^3^	0
	15–25/mm^3^	1
	>25/mm^3^	2
Haemoglobin	>13.5g/dl	0
	11–13.5g/dl	1
	<11g/dl	2
Sodium	<135mmol/l	2
Creatinine	>141μmol/l	2
Glucose	>10mmol/l	1

**Table 2:** The four most common co-morbidities present in patients with necrotising fasciitis

Co-morbidity	Number of patients
Diabetes mellitus	4
Hypertension	6
Obesity	5
Hypercholesteraemia	2

**Table 3:** Site of infection

Site	Number of patients
Arm	5
Leg	6
Scrotum	3
Inguinal	1

**Table 4:** Biomarkers from blood samples on day of admission

Biomarker	Median	Range	Normal range^4^
C-reactive protein (mg/l)	301	68–582	<10
White cell count (× 10^9^/l)	17.8	4.0–38.9	4–11
Haemoglobin (g/dl)	11.8	9.5–15.9	13–18 (men)
11.5–16 (women)			
Sodium (mmol/l)	134	120–142	135–145
Creatinine (μmol/l)	117	83–350	70–150
Glucose (mmol/l)	7.3	2.9–13.4	Fasting: 3.5–5.5

**Table 5:** Microorganisms detected

Type of infection	Microorganism	Number of patients
**Polymicrobial**	Group A *Streptococcus*	2
	Other *Streptococcus*	1 (*Streptococcus milleri*)
	*Staphylococcus aureus*	2 (*Staphylococcus aureus*) 2 (MRSA)
	Gram-negative: other	3 (Mixed anaerobes) 1 (*Escherichia coli* and *Proteus*)
**Monomicrobial**	Group A *Streptococcus*	5
	Other *Streptococcus*	1 (Group G *Streptococcus*)
	*Staphylococcus aureus*	2
	Gram-negative: aerobic	1 (*Klebsiella pneumoniae*)

### Clinical presentation

The median time to presentation after noticing symptoms was 2.0 days (range: 1–7 days). On presentation, seven patients were pyrexial and four were hypotensive. One patient had a rash on the arm. On examination, the findings were erythema, swelling and pain. Five cases presented with frank pus at the site of infection: three had pus in the arm, one in the leg and one in the perineum. The most common site of infection was the lower limb (Table 3.)

### Investigations

Blood samples were taken on the day of admission and the results from the cohort for six variables can be seen in Table 4. Both C-reactive protein (CRP) levels and the white cell count (WCC) were abnormal. Although median haemoglobin levels fell within the normal range for women, it was low in five male patients. Creatinine had a very wide range and was raised in four patients. These six variables were used to calculate the LRINEC score. (See Discussion.)

Five patients had ultrasonography of the infected area and four had computed tomography (CT). All radiographic tests showed subcutaneous oedema and some detected fluid collections. Only in one case, in the CT of the abdomen of the post-panproctocolectomy patient, was gas visible in the soft tissues.

### Microorganisms

Of the 15 patients, 5 had a type I (polymicrobial) infection and 9 had a type II (monomicrobial) infection. One patient showed no evidence of microorganisms in the wound. Table 5 shows the variety of microorganisms detected. The five patients with a polymicrobial infection had a median hospital stay of 109 days (range: 26–131 days) while the nine with a monomicrobial infection had a median stay of 29.0 days (range: 11–60 days).

### Treatment

Nine of the patients underwent debridement within twenty-four hours of admission. Of these, one patient required amputation of the limb during the initial surgery one day after admission. This patient died after 24 days in hospital, after a further 3 operations.

The longest time from presentation to surgery was three days (four patients). One of these four patients died 44 days after admission. The median number of procedures required (including debridements, reviews and skin grafting at later stages) was 3.5 (maximum number was 7). All patients were treated with negative-pressure wound therapy after surgery.

Most patients were treated with broad spectrum antibiotics initially, often before necrotising fasciitis was diagnosed and until the wound sample analysis was received from the laboratory. Five patients were treated on admission for a diagnosis of cellulitis with benzylpenicillin and flucloxacillin, which were altered following the necrotising fasciitis diagnosis in theatre. Other prescriptions included meropenem, vancomycin and linezolid. Of the two patients who had a polymicrobial infection including methicillin-resistant *Staphylococcus aureus* (MRSA), one was treated with vancomycin and piperacillin together with tazobactam while the other received vancomycin, cefuroxime and metronidazole.

The current antibiotic guidelines at our hospital for necrotising fasciitis are 500mg intravenous (IV) metronidazole three times daily, 2g IV amoxicillin three times daily and IV gentamicin once daily. Ciprofloxacin and clindamycin are recommended for patients allergic to penicillin. Group A *Streptococcus* is treated with 1.2g IV clindamycin four times daily.

Two patients received a two-day course of IV immunoglobulins. Both were diagnosed with a Group A *Streptococcus* infection and exhibited severe systemic symptoms such as acidosis and extreme hypotension. One of the patients was given IV immunoglobulins the day after admission, after initial surgery, and had a total hospital stay of 44 days. The other was given IV immunoglobulins after a month in hospital and numerous operations but died 30 days later.

The median duration of stay in hospital was 29 days (range: 11–131 days).

### Mortality

Three of the fifteen patients died (20% mortality rate). The median age of those who died was 68.0 years (range: 60–76 years) while the median age of the rest of the cohort was 48.5 years (range: 34–64 years).

Of these deaths, one patient with severe co-morbidities (8 in all) developed pressure sore areas 11 days after surgical fixation of an ankle fracture. Necrotising fasciitis developed, and was diagnosed and debrided 45 days after the pressure sores were initially noted. The infection was found to be polymicrobial and included MRSA. The second patient had surgery three days after presentation for a Group A *Streptococcus* infection. The third was due to a Group A *Streptococcus* infection in a homeless man with co-morbidities who had suffered a contact burn to his arm and received surgery on the day of presentation.

## Discussion

During the five-year study period, 15 patients were treated for necrotising fasciitis. The median age of the patients was 51.0 years and two-thirds were male. Nine patients had a monomicrobial infection and nine underwent surgical debridement within twenty-four hours of admission. Three patients died from the infection.

Two patients contracted the infection while in hospital, two were transferred with the infection from another hospital and the rest were admitted through the A&E department. The incidence of necrotising fasciitis in the hospital was 0.003%, which is similar to figures for other Western countries, with the number of new cases per year in the UK estimated at around 500.^5^

The mortality rate was 20%. A study in Germany from 2007 reported the mortality rate as 15%^6^ and another showed it was 17% in the US.^7^

There were no obvious predisposing factors such as previous surgery or insect bites in eight cases. All but one patient had co-morbidities, with hypertension, obesity and diabetes mellitus being most common. In theory, hypertension can increase the thrombosis and arteriosclerosis of arteries supplying the skin, predisposing the area to a necrotising infection. Diabetes mellitus is associated with immune deficiencies and arteriosclerosis, both increasing the risk of an infection developing.^8^

The most common infection site was the lower limb and the most common microorganism was a monomicrobial Group A *Streptococcus* infection. Nearly double the number of patients carried a monomicrobial rather than a polymicrobial infection. This is in contrast to other studies, many of which have found more cases of polymicrobial infections,^2^ with one study finding two-thirds of cases were polymicrobial.^9^ Two of our patients were admitted with a polymicrobial infection that included MRSA and required the longest stays in hospital (131 and 117 days respectively). This might attest to the strength of the MRSA infection. There were no cases of monomicrobial MRSA infection although these have been reported over the last decade.^10^

Interestingly, one patient suffered from a monomicrobial disease caused by Group G *Streptococcus*, which is rare. (In fact, only nine cases were reported in the English language literature between 1996 and 2002.)^11^ This patient presented with a four-day history of right leg swelling with multiple blisters and dark erythema. Co-morbidities included hepatitis B, morbid obesity and osteoarthritis. The LRINEC score was only 6 and the patient left hospital after 11 days, having undergone 4 operations.

Another unusual pathogen seen here was monomicrobial *Klebsiella pneumoniae*, which is rare in the Western hemisphere, normally being seen in Asia and the Middle East.^12,13^ There were no obvious co-morbidities in this patient and no recorded history of recent foreign travel.

**Table 6:** Laboratory Risk Indicator for Necrotising Fasciitis score distribution

Score	Risk of infectionbeing necrotisingfasciitis	Number of patients
≤5	Low, <50% chance	3
6–7	Intermediate	6
≥8	High, >75% chance	6

The polymicrobial infections may have been more resilient since in this study the average length of hospital stay was nearly three times that of those patients who had a monomicrobial infection.

In one patient no microorganisms were detected and this patient was treated with broad spectrum empirical antibiotics to cover all possible infections.

The role of radiology studies has been questioned in the past.^14^ In our hospital, they were of no benefit, with only one out of nine studies identifying free gas in the tissues, a sign implicating necrotising fasciitis.

Six biological variables (CRP, WCC, haemoglobin, sodium, creatinine and glucose) were used to calculate the LRINEC score (Table 6). The median of only two of these values (CRP and WCC) fell outside the normal range. The patients in this cohort had a range of scores between 1 and 12, with a median score of 6.5. The median LRINEC score of the three patients who died was 9.0 (range: 6–12).

Three of the fifteen patients would have been categorised as a low likelihood of having necrotising fasciitis. Under these guidelines they would therefore not have received aggressive treatment and surgical debridement.

In the initial study in Singapore, a cut-off score of 6 or above was shown to have a positive predictive value of 92% and a negative predictive value of 96%.^3^ Just over ten per cent of the developmental cohort used in the original study had a score of <6. An explanation for these patients’ low scores could be that they presented very early, before the biomarkers were abnormal, but the records (where available) show that two of these patients had only had one day of symptoms before presentation so this argument is not entirely convincing. The LRINEC system was devised in Singapore so the microorganisms and population may not be readily compared.

Clearly, unless a better scoring system is developed, we think incorporating other factors such as age and comorbidity followed by clinical examination and judgement are the most reliable method of diagnosis.

IV immunoglobulins were given to 2 of the 15 patients. Their use is controversial but is based on the fact that immunoglobulins can block the T cell activation of streptococcal and staphylococcal superantigens. There have been no randomised double blind trials to assess the use of IV immunoglobulins in necrotising fasciitis although they may be of use in septic toxic shock syndrome.^15^ It is not possible to comment on their use in this audit as so few patients were treated.

In this review, it was noted that the whole multidisciplinary team (microbiology, intensivist and surgeon) were involved for swift and effective diagnosis and treatment.

## Conclusions

This five-year review has shown a low incidence of necrotising fasciitis infection with a mortality rate of 20%. Hypertension and obesity were identified as the main co-morbidities. We feel that the LRINEC scoring system alone cannot be treated as an independent method of diagnosis. Emphasis must remain on expert clinical diagnosis and judgement in order not to delay surgical treatment as well as use of the multidisciplinary team.

## Conflicts of interest and source of funding

Ms Beryl De Souza, Dr Berge Azadian, Dr Neil Soni and Dr James Hatcher are all members of staff at the tertiary referral unit involved in this study.
